# Accurate delivery of pristimerin and paclitaxel by folic acid-linked nano-micelles for enhancing chemosensitivity in cancer therapy

**DOI:** 10.1186/s40580-022-00343-5

**Published:** 2022-11-24

**Authors:** Chao Chen, Shiyu Du, Wu Zhong, Kunguo Liu, Lihua Qu, Feiyi Chu, Jingjing Yang, Xin Han

**Affiliations:** 1 Jiangsu Collaborative Innovation Center of Chinese Medicinal Resources Industrialization, School of Medicine & Holistic Integrative Medicine, Nanjing University of Chinese Medicine, Nanjing, 210023 China; 2 Key Laboratory of Study and Discovery of Small Targeted Molecules of Hunan Province, School of Medicine, Hunan Normal University, Changsha, 410013 China; 3 Department of Pathogenic Biology, School of Basic Medical Sciences, Wuhan University, Wuhan, 430071 China

**Keywords:** Pristimerin, Paclitaxel, Nano-micelles, Chemoresistance, Folic acid, Active-targeting

## Abstract

Chemoresistance remains a huge challenge for effective treatment of non-small cell lung cancer (NSCLC). Previous studies have shown Chinese herbal extracts possess great potential in ameliorating tumor chemoresistance, however, the efficacy is clinically limited mainly because of the poor tumor-targeting and in vivo stability. The construction of nano-delivery systems for herbal extracts has been shown to improve drug targeting, enhance therapeutic efficacy and reduce toxic and side effects. In this study, a folic acid (FA)-modified nano-herb micelle was developed for codelivery of pristimerin (PRI) and paclitaxel (PTX) to enhance chemosensitivity of NSCLC, in which PRI could synergistically enhance PTX-induced growth inhibition of A549 cancer cell. PTX was firstly grafted with the FA-linked polyethylene glycol (PEG) and then encapsulated with PRI to construct the PRI@FA-PEG-PTX (P@FPP) nano-micelles (NMs), which exhibited improved tumor-targeting and in vivo stability. This active-targeting P@FPP NMs displayed excellent tumor-targeting characteristics without obvious toxicity. Moreover, inhibition of tumor growth and metastasis induced by P@FPP NMs were significantly enhanced compared with the combined effects of the two drugs (PRI in combination of PTX), which associated with epithelial mesenchymal transition inhibition to some extent. Overall, this active-targeting NMs provides a versatile nano-herb strategy for improving tumor-targeting of Chinese herbal extracts, which may help in the promotion of enhancing chemosensitivity of NSCLC in clinical applications.

**Supplementary Information:**

The online version contains supplementary material available at 10.1186/s40580-022-00343-5.

## Introduction

Malignant neoplasms, such as non-small cell lung cancer (NSCLC), remain one of the leading causes of human health threats [1, 2]. At present, the clinical treatment of malignant tumors mainly includes surgery, radiotherapy or chemotherapy, and immunotherapy, in which chemotherapy shows good tumor suppressive efficiency and is still an indispensable mean of treatment for clinical patients [3, 4]. However, numerous clinical studies have indicated various challenges for effective chemotherapy, including poor tumor-targeting of traditional chemotherapeutic agents and the emergence of chemo-resistance [5, 6]. Chemoresistance of tumor cells make it difficult to achieve effective therapeutic effect of tumors [7]. Additionally, lower accumulation of chemotherapeutic agents in tumor regions result in aborted effective dose and possibility of normal tissue damage. Thus, maximizing efficacy and minimizing side-effects of chemotherapeutic agents through improving the tumor-targeting properties and chemosensitivity are urgently needed.

The anti-tumor effects of natural products, especially Chinese herbal extracts, drive increasing investigations, including ameliorate chemo-resistance. At present, more than 60% of the anti-tumor drugs studied are obtained from natural sources, such as plants, fungi, and microorganisms [8, 9]. Chinese traditional herbs play an auxiliary anti-cancer role by inducing cell apoptosis, enhancing immune system function, and reversing multiple drug resistance [10, 11]. As a natural *quinonemethide triterpenoid* isolated from *celastraceae* and *hippocrateaceae*, pristimerin (PRI) possess multiple pharmacological activities of anti-tumor, anti-inflammatory, and anti-oxidation [12–14]. Studies have shown that PRI exhibits inhibitory effects on a variety of tumors, including lung cancer [15, 16], breast cancer [17, 18], and colorectal cancer [19–21]. Moreover, it has reported that PRI enhances the chemo-sensitivity of paclitaxel (PTX), a classic chemotherapeutic agent, in breast cancer [22] and cervical cancer [23]. However, high-dose of free-drugs (alone or in combination) may induce drug resistance and serious toxic side effects, even though the combination of drugs can be improved to some extent. Thus, further investigating the chemo-sensitization effect and improving the tumor-targeting of PRI in NSCLC is still worth exploration.

Nanodrug delivery-system is presented to achieve the joint delivery of two or more agents, which can overcome the defects of monotherapy and thus realizing synergistic treatment of tumors [24, 25]. Multiple nanodelivery system is developed for tumor-targeting of drugs, such as, liposome, polymeric nanoparticle, and dendrimer [26]. The nanodrug system can ameliorate the physical and chemical properties and enhance vascular penetration of free-drugs to improving the effective doses at the targeting region [27, 28]. In addition, nanodrugs reduce the toxicity of normal cells by a specific modification to achieve its active and passive targeting of the tumor tissues, and realize tumor accumulation through the enhanced permeability and retention (EPR) effect [29, 30]. Studies have demonstrated that combination of nano-herb and chemotherapeutic agents through nanodrug delivery system can further enhance the tumor elimination effects [31–33]. Traditional Chinese medicine monomer has good anti-tumor activity, but its application in tumor treatment is limited by many changes, such as, low solubility and tumor targeting. However, nanocarrier can obtain ideal drug specificity by manipulating the biopharma and pharmacokinetics properties of the molecules, which can largely reduce the characteristics of poor targeting and reduce the adverse toxicity of non-specific distribution. Researchers reflects that the construction of Chinese medicine nano-system shows great effects in the tumor treatment and achieve the characteristics of immune activation [34–36]. Overall, nanodrug delivery system propose a potential strategy for improving the efficacy of tumor chemotherapy in clinic.

**Fig. 1:**
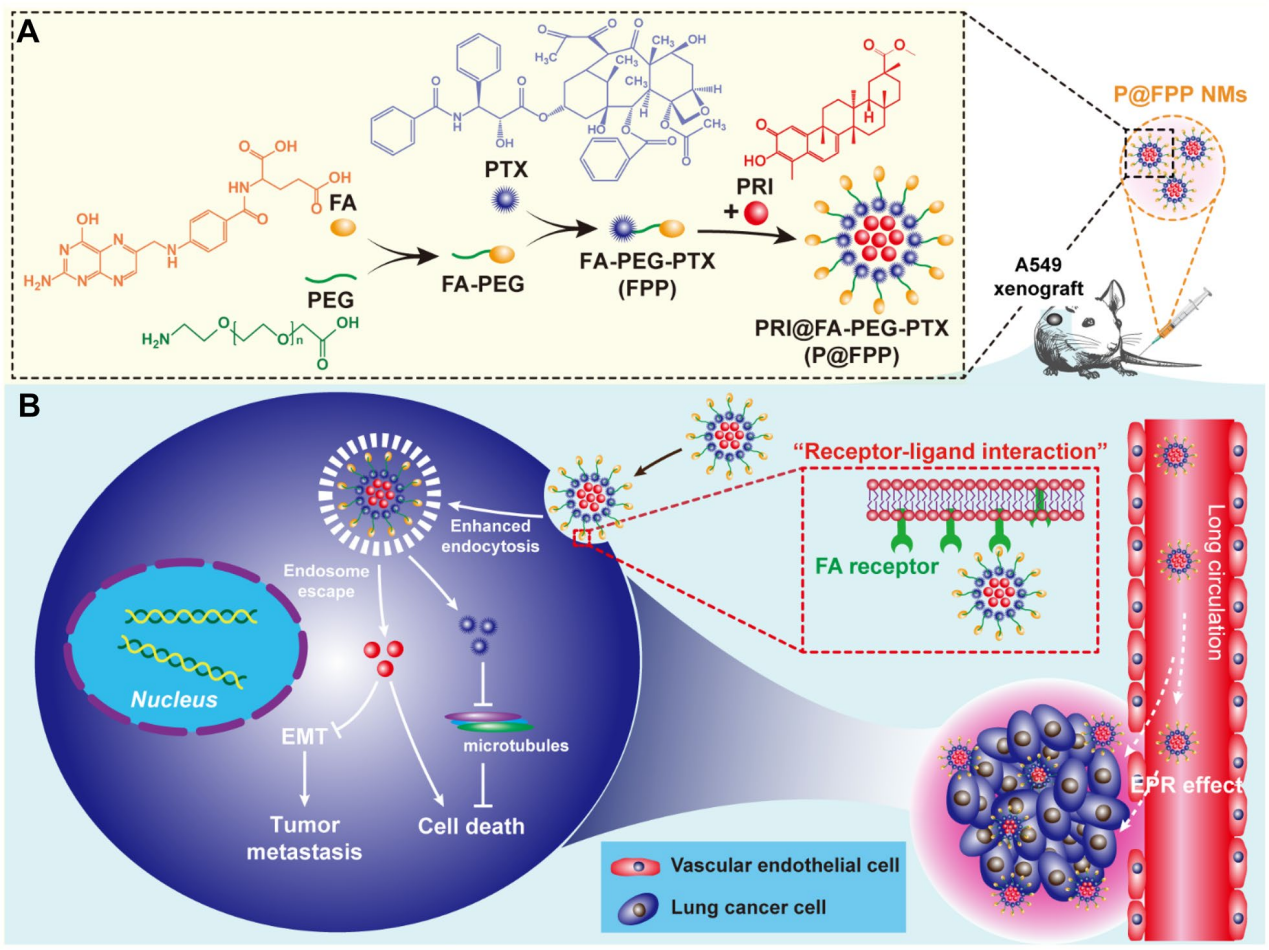
Design of FA-modified nano-herb micelles for codelivery of PRI and PTX. **A** Stepwise synthesis illustration of PRI@FA-PEG-PTX (P@FPP) nano-micelles (NMs). **B** Folic acid (FA) actively targeted NMs co-delivered pristimerin (PRI) and paclitaxel (PTX) for sensitizing chemotherapy of non-small cell lung cancer (NSCLC). P@FPP NMs accumulated in tumor region due to enhanced permeability and retention effect (EPR) and enhanced endocytosis by “receptor-ligand interaction”. PRI and PTX were released from P@FPP NMs after endosomal escape. Subsequently, PRI combined with PTX synergistic inhibited cell viability and metastasis of A549 cells and thus enhanced chemosensitivity of NSCLC

In this study, PRI and PTX were encapsulated in a folic acid (FA)-modified nano-micelles (NMs) to construct the PRI@FA-PEG-PTX (P@FPP) nano-herb (Fig. 1A). The synergistic effect of PRI and PTX in NSCLC of A549 cells was verified in vitro. This simple active-targeting NMs consist of polyethylene glycol (PEG) [37, 38] of skeleton could realize the long-term circulation of plasma and enhanced endocytosis by “receptor-ligand interaction” to improve the chemosensitivity of NSCLC to PTX. The inhibitory effects of P@FPP nano-herb were determined both in vitro and in vivo, which might be related to the epithelial mesenchymal transformation (EMT) phenotypes (Fig. 1B). Meanwhile, P@FPP possessed excellent tumor-targeting capacity with favorable biocompatibility, which providing a novel strategy for nano-herb sensitizing clinical chemotherapy.

## Methods/experimental

### Synthesis of FA-PEG-COOH

1 g of FA and 1 g of EDCI were added into 30 mL DMSO and activated at 40  ℃ for 30 min. After that, 2 g of NH_2_-PEG-COOH was added, and the reaction continued for 2 days. The above reaction solution was dialyzed in deionized water (DI water) with dialysis bag (2500 kDa), and the DI water was changed every hour. The solution was lyophilized two days later to obtain the products.

### Preparation of FA-PEG-PTX

0.15 g of PTX and 0.5 g of CDI were added into 10 mL DMSO, activated at 40 °C for 4 h, with 1 g of FA-PEG-COOH, 0.5 g of EDCI and 0.5 g of DMAP were added into 20 mL DMSO and activated at 40℃ for 30 min. Then, the two reaction solutions were mixed and continued for two days. After that, the above reaction solution was dialyzed in DI water with dialysis bag (2500 kDa), and the DI water was refreshed every hour. The solution was lyophilized two days later to obtain the products.

### ***FTIR and ***^***1***^***H NMR detection***

Fourier Transform Infrared Spectroscopy (FTIR) and Nuclear Magnetic Resonance Spectroscopy (^1^H NMR) were used to test the synthesis of materials. The samples were prepared by taking a small amount of powder, grinding and pressing into pieces with KBr or dissolving in deuterated DMSO. The infrared spectra of samples were obtained by scanning in the range of 500 ~ 4000 cm^−1^.

### Preparation of P@FPP NMs

5 mg of FA-PEG-PTX nano-conjugates were dissolved in 5 mL DMSO. Then, the dialysis bag (7000 kDa) was used to dialysis the above liquid in PBS, and then changed the water every hour. The FA-PEG-PTX was obtained using microporous filter membrane (450 nm) after one day for further detection. 5 mg of FA-PEG-PTX and 0.5 mg of PRI were dissolved in 5 mL DMSO. Then the dialysis bag (7000 kDa) was used to dialysis the above liquid in PBS, and then changed the water every hour. The P@FPP NMs were obtained using microporous filter membrane (450 nm) in the next day.

### Characterization of P@FPP NMs

Dynamic light scattering (DLS) and transmission electron microscopy (TEM) were performed to characterize the structure of NMs. The prepared P@FPP NMs were placed into DTS0012 or DTS1070 cell (Malvern Instruments, Malvern, UK), then placed into the DLS granulometer (DLS, Zetasizer ZS90, Malvern Instruments, Malvern, UK). Each sample was tested three times, 1 min/time. The test conditions were argon ion laser, wavelength 658 nm, temperature 25 ± 0.1 °C, and DLS angle 90°. The ζ-potential was determined at the same time. The operating conditions were 11.4 v cm^−1^, 13.0 mA, and 25 °C. The sample solvent was diluted with distilled water. The NMs samples observed by TEM that were dropped on the copper mesh coated with carbon support film, dyed with 2% phosphoric acid, and dried naturally.

### Drug-loading and release evaluation

The PTX mass fraction was calculated by ^1^H NMR peak area of FA-PEG-PTX and FA-PEG-COOH as follows:

$$\begin{aligned} {\text{Peak}}\,{\text{area ratio (PAR) = }} & \Delta (FA - PEG - {\raise0.7ex\hbox{${{\text{PTX}}}$} \!\mathord{\left/ {\vphantom {{{\text{PTX}}} {{\text{FA}}}}}\right.\kern-\nulldelimiterspace} \!\lower0.7ex\hbox{${{\text{FA}}}$}} \\ & - {\text{PEG - COOH)}}\frac{{{\text{A}}(3.52{\text{pmm)}}}}{{{\text{A(7 - 8}}_{{{\text{ppm}}}} )}} \\ \end{aligned}$$

$${\text{w}}\% \, = { 1}/\left( {{1} + {\text{PAR}}*\left( {{15}*{44}} \right) \, / \, \left( {{4}*{853}.{91}} \right)} \right) \times { 1}00\%$$

The PRI loading and release of NMs were determined by dual wavelength method. FA-PEG-PTX has a peak at the wavelength of 290 nm, as 425 nm of PRI. Both of their absorbances at 290 nm and 450 nm were measured at five of different concentrations to establish standard curve. Diluted the P@FPP NMs and determined the absorbance at two wavelengths, the absorbance of each point is equal to the sum of the absorbance of FA-PEG-PTX and PRI, so the concentration of FA-PEG-PTX and PRI in P@FPP NMs can be calculated. The PTX loading of P@FPP were calculated by the ratio of 10 mg PTX to FA-PEG-PTX at δ = 2.5 ppm. The drug-loading (LC) was calculated as follows:

$${\text{LC}}_{{{\text{PRI}}}} \% \, = \,\frac{{{\text{C}}_{{({\text{PRI}})}} }}{{{\text{C}}_{{({\text{FA}} - {\text{PEG}} - {\text{PTX}})}} + {\text{C}}_{{({\text{PRI)}}}} }}\, \times \,100\%$$

$${\text{LC}}_{{{\text{PTX}}}} \% \, = {\text{c}}_{{{\text{FA}} - {\text{PEG}} - {\text{PTX}}}} \times \, \left( {{1} - {\text{ LC}}_{{{\text{PRI}}}} \% } \right) \, \times {\text{w}}\% \times { 1}00\%$$

To test the drug release of P@FPP, 5 mL of NMs were placed into the dialysis bag (7000 kDa) and dialyzed in 20 mL PBS, shaking on a 37 °C degree shaker. PBS liquid out of the dialysis bag was removed, and 20 mL of fresh PBS was replaced at 0, 0.5, 2, 4, 8, 16, 24 and 48 h. The volume of PBS which has been taken out at each hour was measured accurately and expressed as V_t_, and the concentration of PRI was expressed as C_t_.

The concentration of free-PTX was determined by measuring the absorption at the wavelength of 230 nm with ultraviolet visible spectrophotometer. Since PTX in FA-PEG-PTX released less in PBS with pH 7.4, we use the absorbance of FA to represent the concentration of PTX. The drug release of each time was calculated as follows:

$${\text{Q}}_{{\text{t}}} \% \,\, = \,\frac{{\sum\nolimits_{{\text{t = 0}}}^{{\text{n}}} {{\text{V}}_{{\text{t}}} {\text{C}}_{{\text{t}}} } }}{{{\text{V}}_{{{\text{db}}}} {\text{C}}_{{\text{db(PRI)}}} }}\, \times 100\%$$

where Q_t_ denotes the drug release rate at t hour, **V**_**db**_ represents the volume of PBS in the dialysis bag, **C**_**db(PRI)**_ is the initial concentration of the NMs sample. (t = 0, 0.5, 2, ⋯, n, ⋯, 48 h, both V_0_ and C_0_ are equal to zero).

### Endocytosis verification of P@FPP NMs

To evaluate the cell uptake of P@FPP NMs by A549 cells, the fluorochrome of Indocyanine green (ICG) were used to instead PRI for constructing ICG@FPP (I@FPP) NMs. In brief, A549 cells (1 × 10^5^) were plated and incubated with I@FPP for 2, 4, 6 h, and then the fluorescence under the fluorescence microscope were observed and imaged after nucleus staining with Hoechst 33342.

### Cell culture and cell viability evaluation

#### Cell culture

Human non-small cell lung cancer (NSCLC) of A549 cells were obtained in house, but are available from American Type Culture Collection (ATCC). A549 cells were cultured in Dulbecco’s modified Eagle’s medium (DMEM; HyClone) supplemented with 10% fetal bovine serum (FBS; Gibco) and 1% penicillin–streptomycin in a humidified atmosphere of 95% air and 5% CO_2_ with the temperature of 37 °C.

#### Cell viability evaluation

To detect the effects of free-drugs or NMs on cell viability, the A549 cells was dispersed into signal-cell suspension and seeded at a density of 4 × 10^4^ cells/well in 96-well plates for 18–24 h, then, the cells were treated with free-drugs or P@FPP NMs for 48 h. After that, 10 µL of Cell Counting Kit-8 kit (CCK8, YIFEIXUE BIO TECH) was added into each well. The optical density at 450 nm was examined by a Multimode Plate Reader (EnVision, PerkinElmer) after incubation of 60 min.

$${\text{Cell viability }}\left( \% \right) \, = \frac{{\left[ {{\text{OD}}_{{{49}0 \, ({\text{treated groups}})}} - {\text{ OD}}_{{{49}0 \, ({\text{background}})}} } \right]\,}}{{\left[ {{\text{OD}}_{{{49}0 \, ({\text{control groups}})}} - {\text{ OD}}_{{{49}0 \, ({\text{background}})}} } \right]}} \, \, \times { 1}00\% .$$

### Cell migration and invasion assays

Cell migration and invasion of A549 cells were determined using Transwell system (Corning) with or without Matrigel (Corning). A549 cells were administrated with free-drugs or P@FPP NMs for 24 h and then performed Transwell assay. Briefly, cells with density of 5 × 10^4^ (for migration) or 1 × 10^5^ (for invasion) in serum-free culture medium were seeded into the upper chamber and medium containing 10%-serum was added to the bottom chamber. After incubation at 37 °C for 24 h, the noninvading cells in the upper chamber were removed scrubbing, and the migrating or invading cells in the bottom chamber were fixed with 4% paraformaldehyde and then stained with 0.1% crystal violet solution. The cells were observed and photographed under the light microscope.

### Quantification real-time PCR

Total RNA was extracted using the RNA isolater Total RNA Extraction Reagent (Vazyme), and 1 μg was used to reverse-transcribe cDNA. The resulting cDNA was used as a template for quantitative PCR in 20 μL reactions containing 2 μL of cDNA, 0.4 μL of a forward primer, 0.4 μL of a reverse primer, 7.2 μL of ddH_2_O, and 10 μL of 2 × ChamQ Universal SYBR qPCR Master Mix (Vazyme). The temperature program (95 °C for 10 s, 58 °C for 30 s, and 72 °C for 30 s) was repeated 40 times. The primers used to quantify epithelial-mesenchymal transition (EMT)-related genes (E-cadherin, N-cadherin, Vimentin, and Twist) were listed in Additional file 1: Table S1.

### Western blot analysis

The whole cell lysates were obtained from the treatment cells by using 1 × cell lysis buffer (Cell Signaling Technology) with 1 mM phenylmethanesulfonyl fluoride (Sigma-Aldrich) and 1 × protease inhibitor cocktail (Roche). Then, the whole cell lysates were collected and quantified using the BCA protein quantification method. the protein samples were mixed with the loading buffer and denatured by boiling and electrophoresis on 8 or 10% denaturing PAGE gels followed by incubation of the corresponding primary antibodies (Additional file 1: Table S2) and the HRP-conjugated secondary antibodies. After that, the protein bands were visualized using ChemiDoc XRS + with Image Lab software (Bio-Rad).

### In-vivo biodistribution and xenograft inhibition of P@FPP NMs

#### In-vivo biodistribution of P@FPP NMs

To investigate the biodistribution of P@FPP, we first assembled fluorochrome of ICG-loading I@FPP NMs, and then the A549 tumor-bearing nude mice were intravenously injected with I@FPP. The fluorescence distribution in vivo was monitored 6 h after injection by IVIS system (PerkinElmer). Subsequently, the mice were euthanized and the major organs (heart, liver, spleen, lung, and kidney) and tumor tissue were dissected and the fluorescence distribution were monitored.

#### Xenograft inhibition of P@FPP NMs

The A549 xenograft bearing nude mice (~ 80 mm^3^) were randomly divided into five groups (n = 4) and then intravenously injection of the following formulations: PBS, PRI, PTX, PRI in combination of PTX, and P@FPP NMs. The mice were administrated every two days for 20 days. The mouse weight and tumor volume were monitored in the treatment process, and the tumor volume was calculated as the following formula: volume = 0.5 × (length × width^2^). The mice were euthanized and then, the tumor tissues and the major organs were harvested at the end of experiment for further analysis. All animal procedures were performed under the guidance of the Animal Care and Use Committee of Nanjing University of Chinese Medicine (ethical approval number: 202204A031).

### Histology analysis

The obtained tissues were fixed and embedded with 4% paraformaldehyde and paraffin, respectively, and then cut into 4-µm-thick sections for H&E staining. For immunohistochemical analysis, the tissue sections were preformed according to the manufacturer’s instructions. Briefly, the tumor sections were first stained with the monoclonal anti-E-cadherin, anti-N-cadherin, anti-Ki-67, and TUNEL, and then stained with 3,3′-diaminobenzidine (DAB) and counterstained with hematoxylin. After that, the sections were observed under bright-field microscope (DMi 8, Leica). The cells with brown granules were considered as the positively stained cells.

### Statistical analysis

All the experiments were repeated three times. The data represented as mean ± standard deviation (SD). To ascertain the significance of the differences between the mean values of the different experimental groups, one-way ANOVA was employed followed. *P* < 0.05 and *P* < 0.01 were considered to be significant while *P* < 0.001 was considered to be highly significant.

## Results and discussion

### *Pristimerin enhances chemosensitivity of PTX to A549 cells *in vitro

**Fig. 2:**
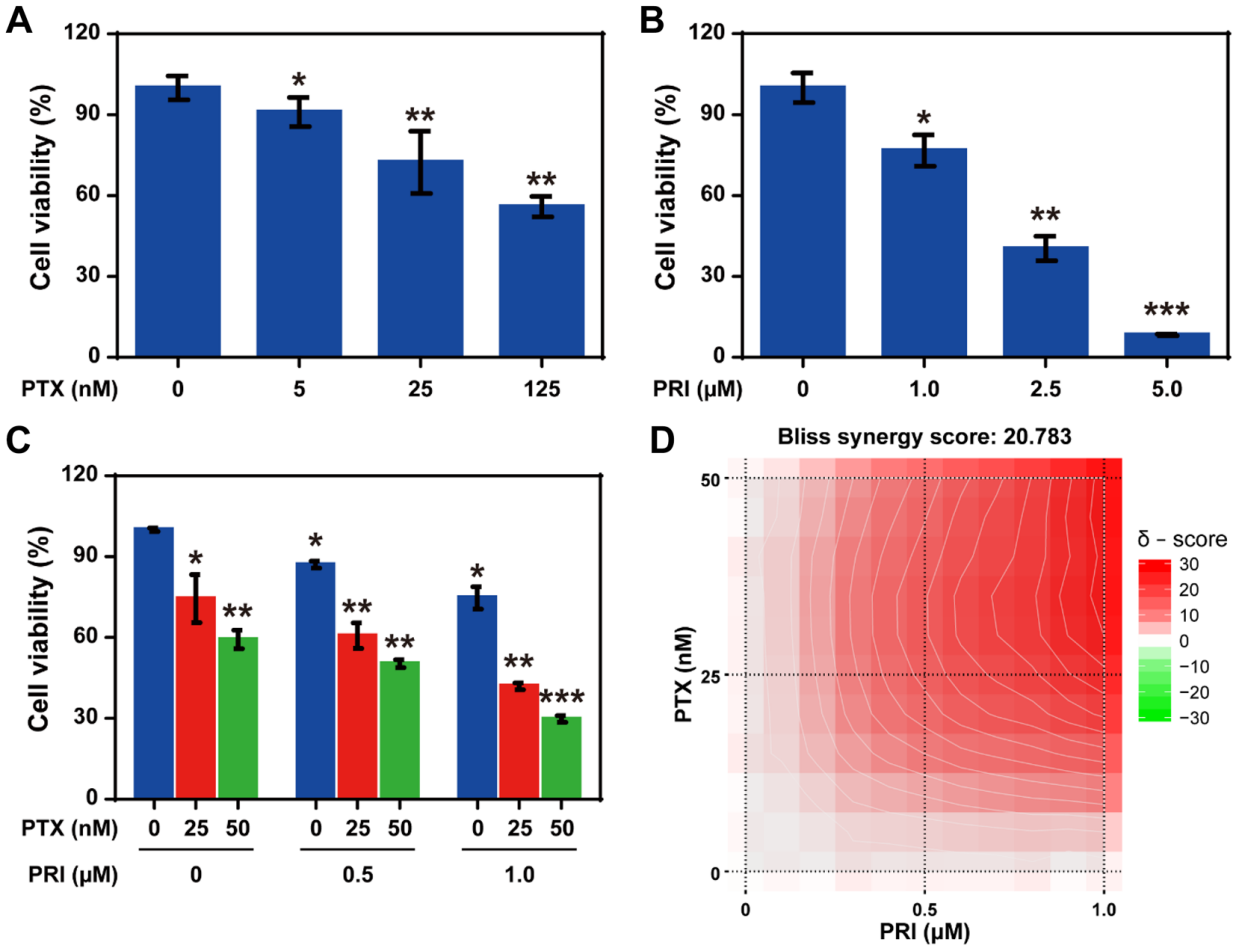
Synergistic inhibitory effects of PRI and PTX on cell viability of A549 cells. **A** Cell viability of A549 cells treated with different concentrations of PTX (n = 3). **B** Inhibition of cell viability on A549 cells treated with PRI (n = 3). **C** A549 cells treated with PTX alone or in combination of PRI (n = 3). **D** The synergistic score of PTX combined with PRI calculated by using the Synergyfinder 2.0 software. *: *P* < 0.05. **: *P* < 0.01. ***: *P* < 0.001

The cell viability inhibition of pristimerin (PRI) and paclitaxel (PTX) against non-small cell lung cancer (NSCLC) of A549 cells were detected by CCK-8 assays. We first analyzed the cytotoxicity of free-drugs (free-PTX and free-PRI) on A549 cells (Fig. 2A, B). The results shown that both of PRI and PTX revealed a concentration-dependent inhibition of cell viability, and the half maximal inhibitory concentration (IC_50_) values of PTX and PRI on A549 cells were 174.6 nM and 1.91 µM, respectively. Moreover, to evaluate the synergistic effects of PTX and PRI, A549 cells were treated with PTX alone or in combination with PRI. As descript in Fig. 2C, the cell viability inhibitory efficiency of A549 cells by 25 nM of PTX in combination of 1.0 μM of PRI was 58.1% ± 1.2%, which higher than that of the superposition of 25.5 ± 8.8% for 25 nM of PTX and 25.3 ± 4.1% for 1.0 μM of PRI. Meanwhile, the combination of 50 nM of PTX and 1.0 μM of PRI revealed the inhibitory effects of 70.2% ± 1.3%, which also stronger than the arithmetical overlay of respectively 40.7 ± 3.5% for 50 nM of PTX and 25.3 ± 4.1% for 1.0 μM of PRI (Fig. 2C).

Furthermore, the correctional Bürgi formula [q = E (A + B)/(EA + EB – EA·EB)] was employed to further confirm the synergistic effects of PTX in combination of PRI, and in which E (A + B) for the combination inhibitory effect, EA and EB respectively for the individual inhibitory effect, and q ≥ 1 indicated the synergistic effect of two drugs. The acquired results demonstrated that the q values of four combination groups for PTX and PRI respectively were: q = 1.12 for 0.5 μM PRI + 25 nM PTX, q = 1.31 for 0.5 μM PRI + 50 nM PTX, q = 1.03 for 1.0 μM PRI + 25 nM PTX, and q = 1.26 for 1.0 μM PRI + 50 nM PTX, which both suggesting synergistic effects of PTX combined with PRI (q ≥ 1). In addition, the Synergyfinder 2.0 software was next used to analyze the synergistic effects of two drugs [39]. As depicted in Fig. 2D, the synthesis score of PRI in combination of PTX was 20.783, indicating that PRI combined with PTX revealed synergistic inhibitory effect on A549 cells within the studied dose range. In summary, PRI can significantly enhance chemosensitivity to PTX in NSCLC of A549 cells.

### Preparation and characterization of P@FPP NMs

**Fig. 3:**
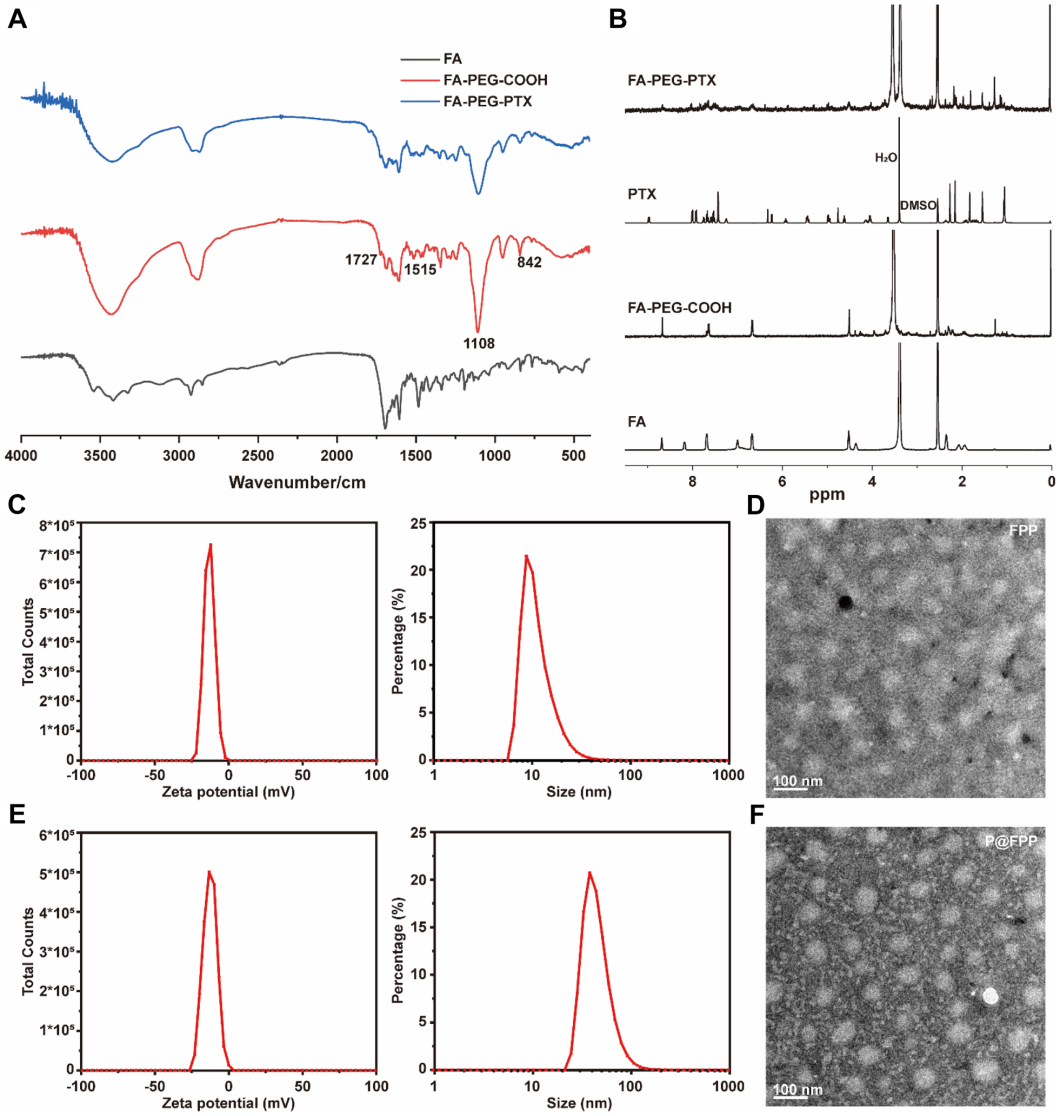
Preparation and characterization of P@FPP NMs. **A** The Fourier Transform Infrared Spectroscopy (FTIR) analysis of FA, FA-PEG-COOH, and FA-PEG-PTX. **B** The ^1^H Nuclear Magnetic Resonance Spectroscopy (^1^H NMR) analysis of FA, PTX, FA-PEG-COOH, and FA-PEG-PTX. **C** The size distribution and ζ-potential of FA-PEG-PTX. **D** The representative transmission electron microscopy (TEM) image of FA-PEG-PTX. Scale bars: 100 nm. E The size and ζ-potential analysis of P@FPP NMs. **F** The TEM observation of P@FPP. Scale bars: 100 nm

Encouraged by PRI in combination of PTX synergistically inhibited the cell viability of A549 cells, we assembled a folic acid (FA)-modified activate-targeting nano-herb micelle PRI@FA-PEG-PTX (P@FPP) and the synthetic process as provided in Fig. 1A. First, to evaluate whether the FA-PEG-PTX nano-conjugates were successful constructed, the Fourier Transform Infrared Spectrogram (FTIR) and ^1^H Nuclear Magnetic Resonance (^1^H NMR) were detected. As depicted in Fig. 3A, B, compared with FA, FA-PEG-COOH had ether bond C–O–C stretching vibration peak at 1108 cm^−1^, para substituted benzene ring stretching vibration peak at 842 cm^−1^, benzene ring double bond stretching vibration peak at 1515 cm^−1^, C = N stretching vibration peak at 1693 cm^−1^, polyamide carbonyl stretching vibration peak at 1727 cm ^−1^, the 3.52 ppm -CH_2_CH_2_- peak of PEG, and 7–8 ppm peaks on benzene ring. All of this indicated that the FA-PEG-COOH was synthesis successfully. Meanwhile, FA-PEG-PTX had a C = O stretching vibration peak in paclitaxel and its PEG binding site at 1793 cm^−1^, and on the NMR spectrum, the peak of the paclitaxel benzene ring at 7–8 ppm appears on the FA-PEG-PTX, as so as the characteristic peak of paclitaxel at 5–6.5 ppm, which showed successful grafting of PTX.

After PRI was successful packaged by FA-PEG-PTX nano-conjugate, we then detected the size, ζ-potential, and morphology of the P@FPP nano-micelles (NMs). The results indicated that the size of the FA-PEG-PTX was 69.25 ± 5.74 nm (PDI = 0.278), and the ζ-potential of the FA-PEG-PTX was -13.1 ± 0.64 mV, and shown a good dispersion under transmission electron microscopy (TEM) observation (Fig. 3C, D). After successful loaded with PRI, the size distribution and ζ-potential of P@FPP were 104.6 ± 4.84 nm (PDI = 0.236) and − 12.7 ± 0.38 mV, respectively (Fig. 3E), and TEM images suggested favorable dispersion and uniform size (Fig. 3F). In conclusion, both of the FA-PEG-PTX nano-conjugates and P@FPP NMs have a small PDI and a highly surface potential, which leaded to a stable quality. The P@FPP indicated larger size and lower ζ-potential than that of the nano-conjugates, suggesting that the P@FPP NMs were successfully prepared.

### In-vitro drug-loading and drug release of P@FPP NMs

To detect the drug-loading efficiency of our synthesized P@FPP NMs, we measured the drug loading of the NMs and calculated according to the formula, the PRI loading was 11.56 ± 0.08% while the drug loading of PTX was 4.06 ± 0.54% (Additional file 1: Fig. S1). Because of the relatively simple structure and the relatively small molecular weight of polyethylene glycol (PEG) we used, the drug loading of P@FPP NMs should be relatively high by bonding with some drug and encapsulating another drug. These above results shown that the P@FPP have a good drug-loading capacity. Overall, this kind of NMs reduce the use of nanomaterials, reduce waste, while improve the effective concentration and the curative effects.

**Fig. 4:**
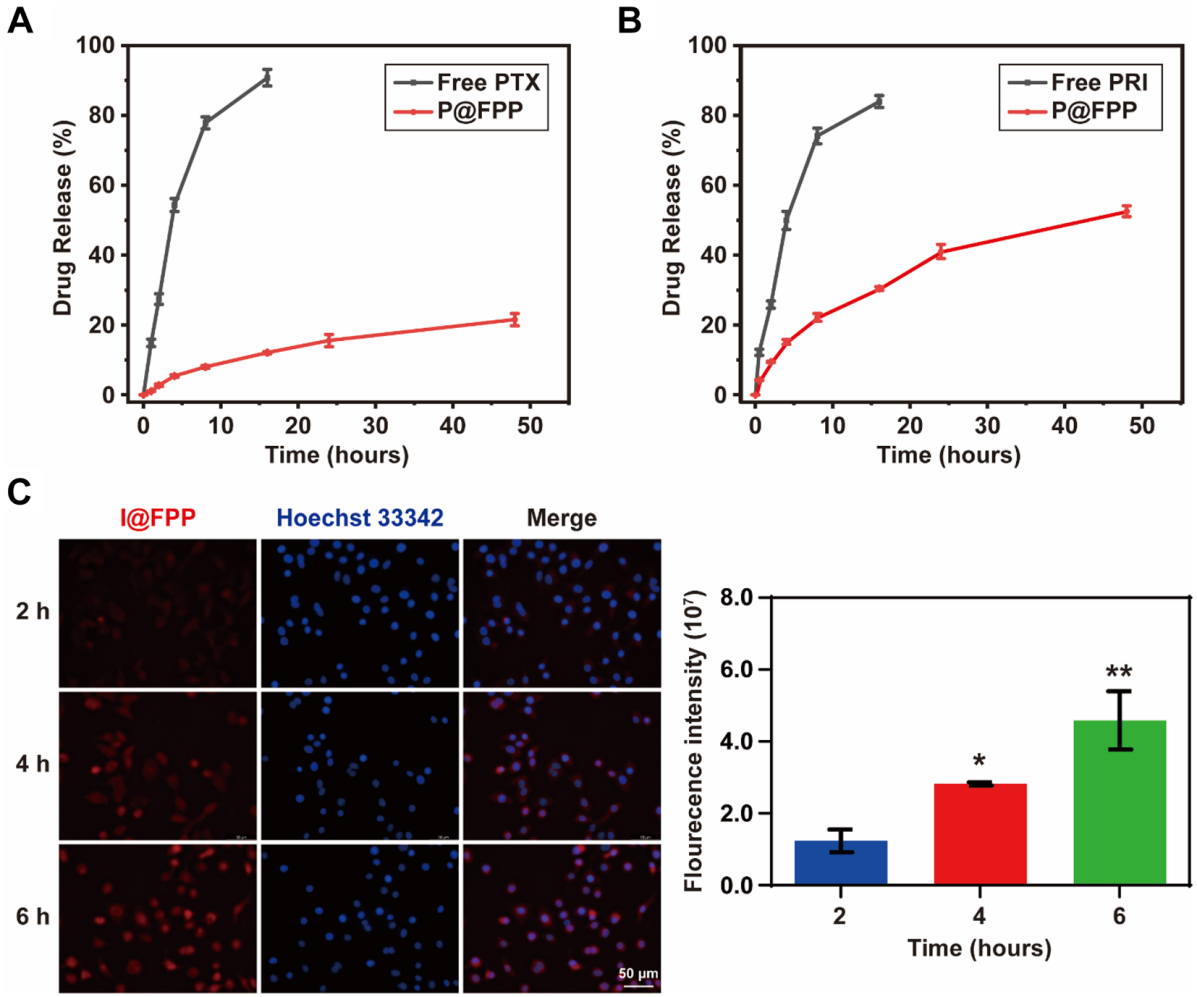
In-vitro release profiles of drugs from P@FPP NMs. **A** The release of PTX from P@FPP NMs in phosphate buffered saline (PBS) at different times (pH = 7.4). **B** The drug release of PRI from P@FPP. **C** Cellular uptake of A549 cells after treated with ICG-loading NMs (I@FPP) for 2, 4, and 6 h observed by using fluorescence microscope (n = 3). Red, ICG; Blue, Hoechst 33,342. Scale bar: 50 μm. *: *P* < 0.05. **: *P* < 0.01

As shown in Fig. 4A, the FA-PEG modification reduced the drug release of PTX. The PTX release from P@FPP NMs were significant lesser than that of the free-PTX. The release rate of free-PTX was 14.88 ± 1.07%, while that of P@FPP was only 1.02 ± 0.10% in 0.5 h. 90.78 ± 2.41% of free-PTX was released in 16 h, but even after 48 h, there only 21.52 ± 1.77% of PTX in P@FPP was released. Meanwhile, the nano-encapsulation of drugs can also effectively slow down the release of PRI. The release rate of free-PRI was 12.19 ± 0.90%, while that of the NMs was only 4.20 ± 0.17% in 0.5 h. And in 16 h, most of the free-PRI had been released, accounting for 83.97 ± 1.69%, while 30.44 ± 0.54% of the F@FPP. After 48 h, the release rate of F@FPP raised to 52.54 ± 1.55%, suggesting half of the PRI was still retaining (Fig. 4B). Thus, it could be considered that P@FPP NMs greatly slowed down the release of the two drugs, indicating the lower release rate would further improve the effect in biological experiment. To further detect the endocytosis of NMs in tumor cells, ICG@FPP (I@FPP) NMs were constructed with fluorochrome of indocyanine green (ICG) instead of PRI, and then the nuclei was stained with Hoechst 33342. As depicted in Fig. 4C, cell uptake experiments were employed to evaluate the cellular internalization of NMs. Within 6 h of the experiment, the cellular uptake of NMs increased gradually, showing that NMs caused more sustained cellular uptake. The above results indicated that P@FPP NMs displayed controlled release of PTI and PTX while promoted the endocytosis of tumor cells.

### *Cell viability and metastasis inhibition of P@FPP NMs *in vitro

**Fig. 5:**
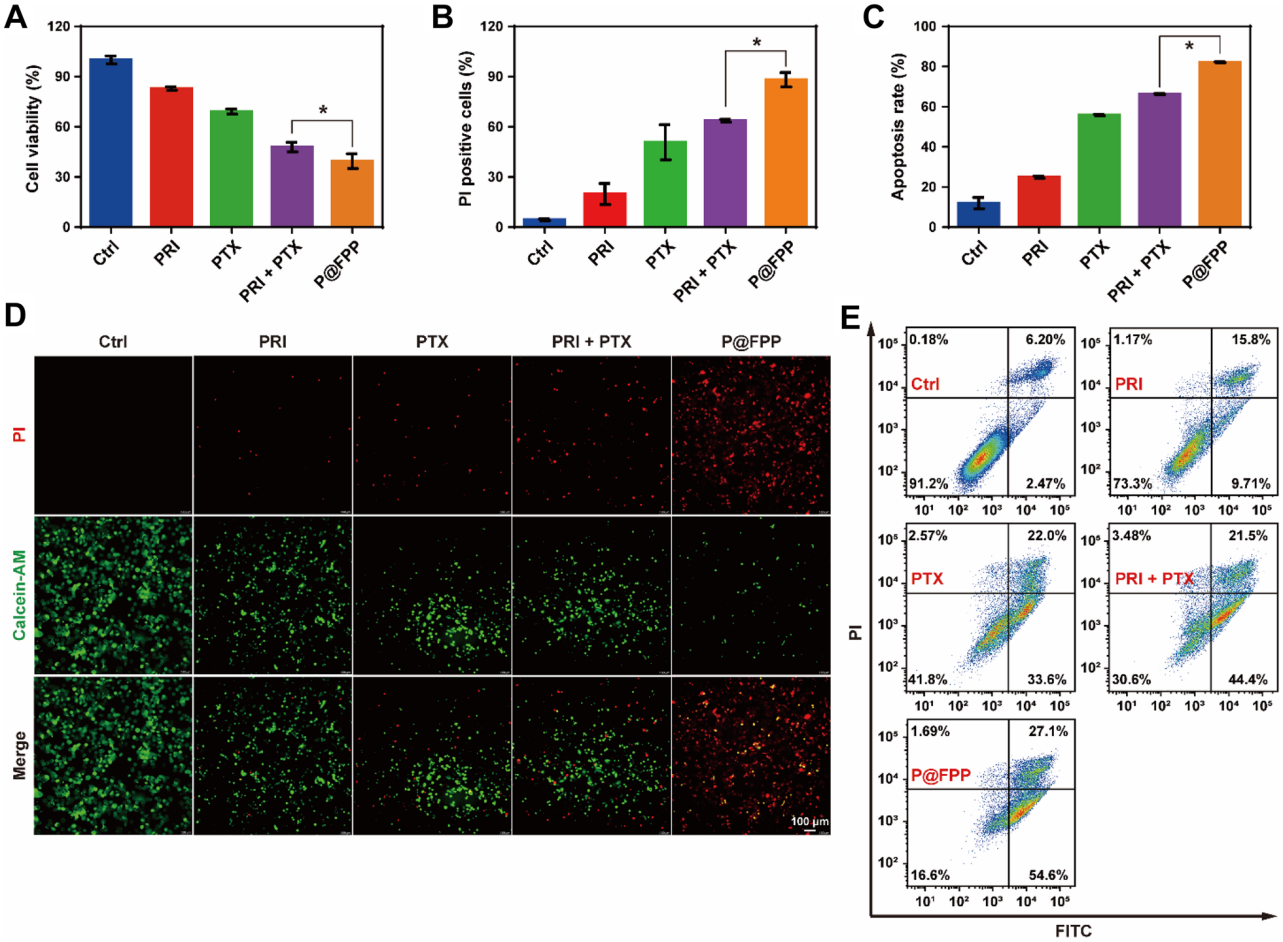
In vitro cell viability inhibition of P@FPP NMs. **A** The cell viability of A549 cells detected by CCK8 assay after treatment of PBS, PRI, PTX, PRI + PTX, and P@FPP (n = 3). **B** The cell death analysis of A549 cells administrated with different treatments using Calcein-AM/PI staining assays (n = 3). **C** Flow cytometry analysis for cell apoptosis of A549 cells treated with different formulations (n = 3). **D** The representative fluorescent images of tumor cells by treatment of different treatments. Green, Calcein-AM; Red, PI. Scale bar: 100 μm. **E** Representative images for flow cytometry of A549 cells with different treatments. *: *P* < 0.05

After successful assembly and characterization of P@FPP NMs, the cell viability inhibition of A549 cells after treatment of P@FPP were determined in vitro. A549 cells were first administrated with different concentrations of F@FPP and monitored by using CCK8 assay. The results indicated that F@FPP NMs could obviously inhibit the proliferation of A549 cells in a concentration-dependent manner, and an excellent inhibitory efficiency (80.9 ± 1.1%) was obtained at the concentration of 50 μg/mL (Additional file: 1 Fig. S2). Moreover, compared to PRI combined with PTX treatment (52.9 ± 2.8%), P@FPP NMs exhibited significant further inhibition of cell viability (60.5 ± 4.4%) in A549 cells (Fig. 5A). The results of live/dead fluorescent cell staining detection indicated that tumor cells with incubation of P@FPP NMs showing the highest red intensity (indicated dead cells) and the weakest green intensity (showed live cells) (Fig. 5B and D. To further confirmed the inhibitory effects of P@FPP on A549 cells, flow cytometry analysis was used to detect the apoptosis induction of P@FPP. As shown in Fig. 5C and E. compared with treatment of PRI or PTX alone, PRI in combination of PTX increased the apoptosis ratio of A549 cells, and P@FPP could further enhance the cell apoptosis. Overall, these results indicated the synthesized NMs could effective inhibited A549 cells in vitro.

**Fig. 6:**
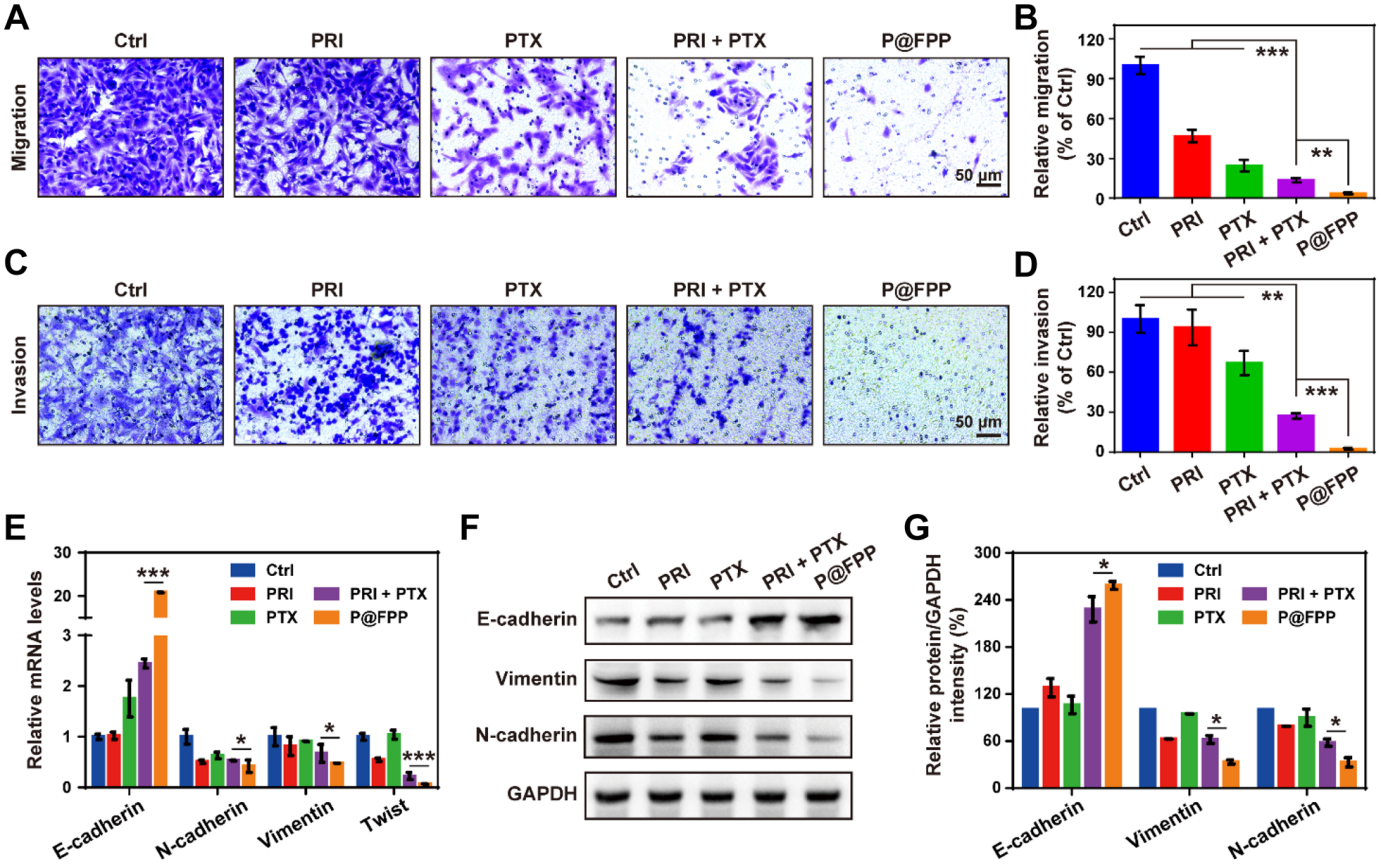
Cell migration and invasion inhibition of P@FPP NMs in vitro. **A–B** Representative cell migration images and corresponding quantitative analysis of A549 cells treatment of PBS, PRI, PTX, PRI + PTX, and F@FPP NMs using Transwell assay (n = 3). **C–D** Cell invasion analysis of tumor cells with different formulations (n = 3). **E** The mRNA levels of epithelial-mesenchymal transition (EMT) related genes (E-cadherin, N-cadherin, Vimentin, and Twist) in A549 cells with different formulations by using qRT-PCR analysis (n = 3). **F–G** Western blot and relative quantitative analysis of E-cadherin, N-cadherin, and Vimentin expression in 549 cells after different treatments (n = 3). *: *P* < 0.05. **: *P* < 0.01. ***: *P* < 0.001

Studies have reported that PRI can inhibit migration and invasion of various tumor cells [14, 19], including NSCLC cells [40]. The effects of F@FPP on A549 cell migration was tested by using scratch test and Transwell system, and it was indicated that the migration capacity of tumor cells with treatment of P@FPP NMs was obviously inhibited compared to the PRI combined with PTX administration (Fig. 6A, B and Additional file 1: Fig. S3). The invasion ability of A549 cells was also detected by using Transwell system, and the results shown that P@FPP exhibited the highest inhibition of cell invasion among all experimental groups (Fig. 6C, D). These results suggested that P@FPP NMs proposed a robust inhibitory effect on migration and invasion of tumor cells. In addition, it has reported that Epithelial-mesenchymal transition (EMT) is a key molecular mechanism of tumor metastasis. To verify whether cell migration and invasion inhibition by P@FPP was associated with EMT process, qRT-PCR analysis and Western blotting were performed to evaluate the mRNA and protein levels of EMT related genes, respectively. As depicted in Fig. 6E, P@FPP NMs increased the mRNA levels of E-cadherin, and decreased the levels of N-cadherin, Vimentin, and Twits in A549 cells, compared with PRI combined with PTX. Meanwhile, the conclusions of EMT-related protein (E-cadherin, N-cadherin and Vimentin) expression by Western blot analysis were consistent with the results of qRT-PCR analysis (Fig. 6F, G), i.e., P@FPP NMs upregulated the protein level of E-cadherin, and downregulated the protein expression of N-cadherin and Vimentin. In summary, inhibition of migration and invasion by P@FPP may be related to the EMT phenotypes, to some extent.

### In-vivo xenograft and EMT phenotype inhibition of P@FPP NMs

**Fig. 7:**
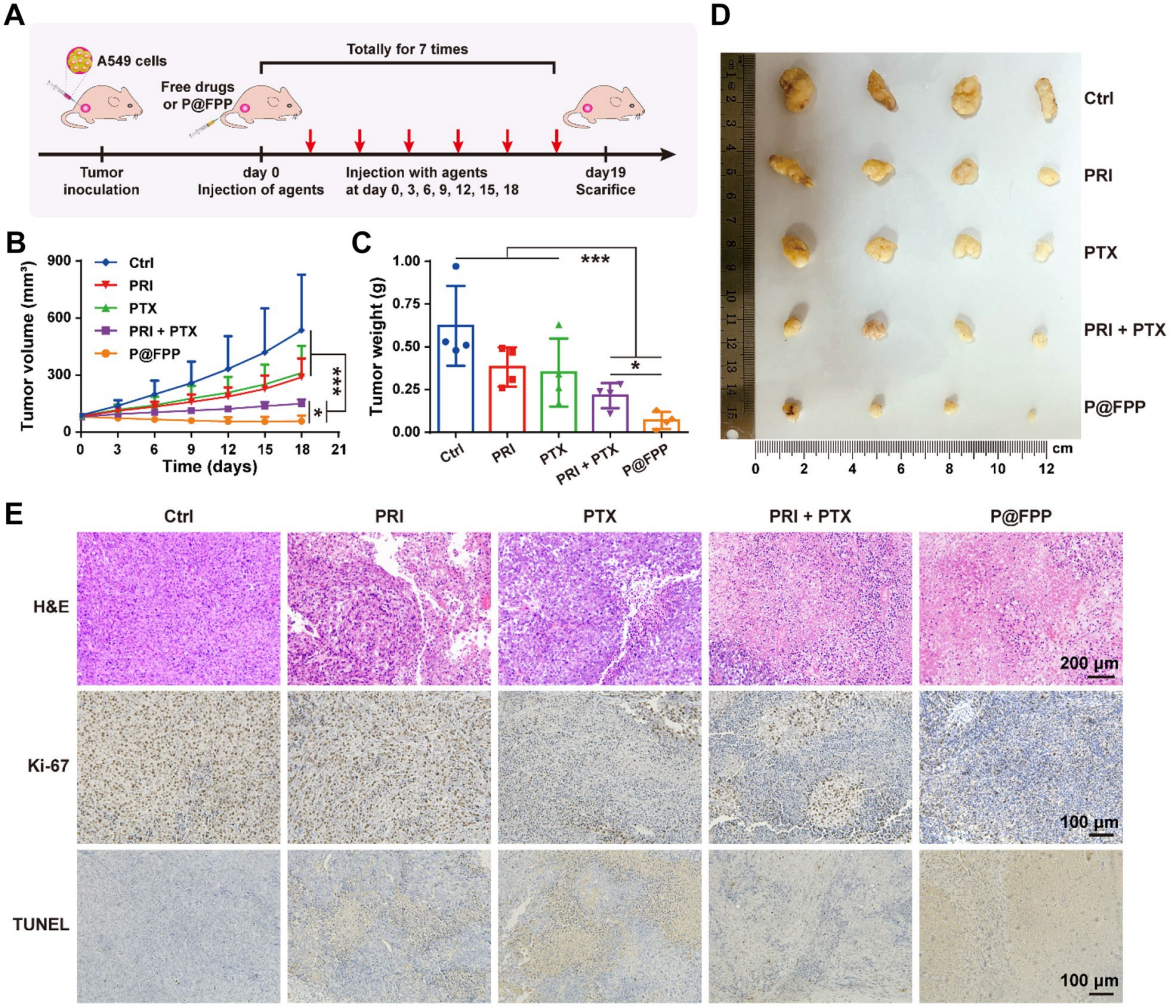
Tumor growth inhibition of P@FPP NMs in vivo.** A** Schematic illustration of the treatment process in nude mice bearing tumor. **B** The changes of tumor volumes under different treatment process (n = 4). **C** The tumor weights under treatments of different formulations (n = 4). **D** The physical images of tumors treated with PBS, PRI, PTX, PRI + PTX, and P@FPP. **E** Representative images of H&E staining, IHC for Ki-67, and TUNEL (terminal deoxynucleotidyl transferase–mediated deoxyuridine triphosphate nick end labeling) staining of tumor tissues. scale bar of H&E staining: 200 μm. scale bar for IHC of Ki-67 and TUNEL staining: 100 μm. *: *P* < 0.05. ***: *P* < 0.001

Inspired by the favorable results in vitro, we continued to explore the antitumor activities of P@FPP NMs in xenograft model. The Balb/c mice bearing xenograft of A549 cells (~ 80 mm^3^) were randomly divided into five groups and administration of different formulations via intravenous injection every two days (Fig. 7A). The tumor volumes of all experimental groups were monitored in the next 20 days (Fig. 7B). The inhibition of tumor growth was significantly stronger in P@FPP NMs than that of the other groups including PRI in combination of PTX, showing almost xenograft elimination in mice. The tumor tissues were harvested and weighed after euthanasia of mice at the end of experiment (Fig. 7C, D), which further confirmed the robust inhibition of xenograft growth. Moreover, the histologic analysis was performed in the tumor sections of mice. As provided in Fig. 7E, A decreased cell density in groups with the treatment of PRI, PTX, PRI combined with PTX, and P@FPP were observed under the hematoxylin and eosin staining of tumor slices, compared to the PBS treated group. The in-situ Ki-67 and TUNEL staining exhibited an obviously reduction of cell proliferation and induction of cell apoptosis, respectively, after treatment of PRI, PTX, PRI in combination of PTX, and P@FPP, especially for P@FPP NMs administration. Overall, our assembled P@FPP NMs showed a robust inhibition of xenograft.

**Fig. 8:**
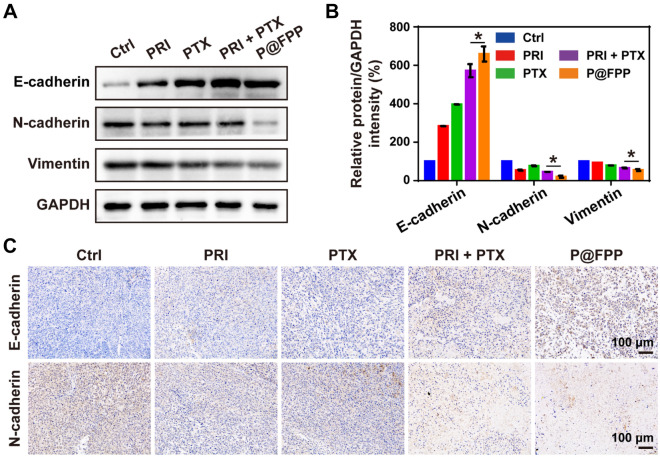
EMT inhibition of P@FPP NMs in vivo. **A–B** Western blot and corresponding quantitative analysis of E-cadherin, N-cadherin, and Vimentin protein levels in tumor tissues (n = 3). **C** IHC for E-cadherin an N-cadherin of tumor tissues. scale bar: 100 μm. *: *P* < 0.05

The EMT phenotypes inhibition was also confirmed in the tumor tissues. As shown in Fig. 8A, B, the protein expression of EMT-related genes (E-cadherin, N-cadherin, and Vimentin) were detected by Western blot analysis, and the results shown that compared with the PRI in combination of PTX group, P@FPP could further upregulated the protein level of E-cadherin and downregulated the levels of N-cadherin and Vimentin. Additionally, immunohistochemical analysis of EMT-related proteins (E-cadherin and N-cadherin) of tumor tissues approved the results of Western blotting, showing the highest expression of E-cadherin and lowest expression of N-cadherin were observed in the tumor sections of P@FPP treatment group (Fig. 8C). The above data indicated that P@FPP inhibited EMT process in vivo.

### In-vivo biosafety evaluation of P@FPP NMs

**Fig. 9:**
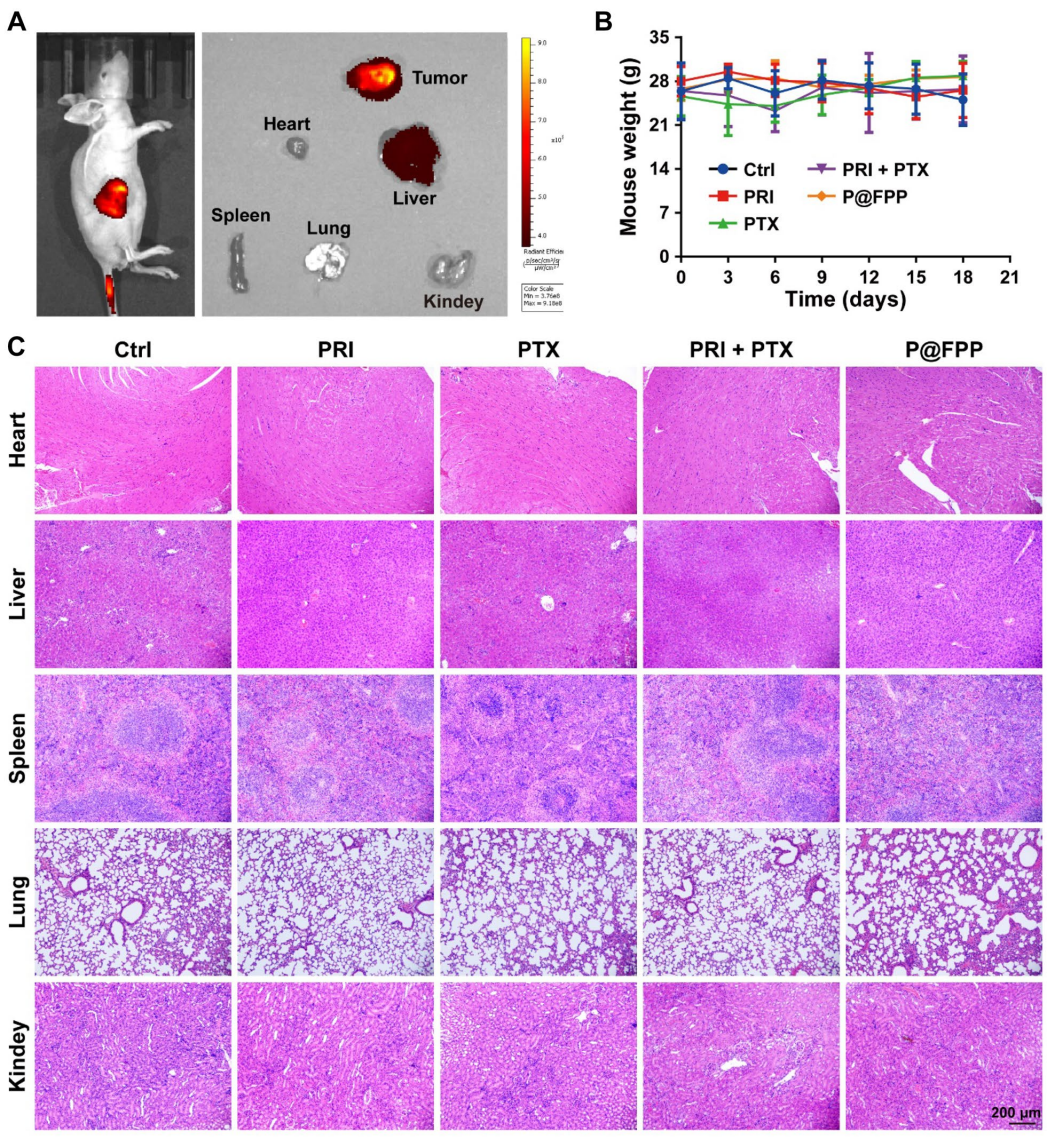
In vivo biosafety evaluation of P@FPP NMs. **A** In vivo fluorescence imaging after intravenous injection with the ICG@FA-PEG-PTX (I@FPP) NMs at 4 h. **B** Body weight curves of A549 tumor-bearing mice with different treatments. **C** Representative images of H&E staining of major organs of tumor-bearing mice. Scale bar: 200 μm

Nano-herb delivery system with clinical transformation potential needs not only robust therapeutic efficacy, but also highly biosafety. Firstly, we employed ICG to assemble the I@FPP for evaluating the biodistribution of NMs in vivo, and demonstrated that the I@FPP was highly accumulation at the tumor region via IVIS imaging, suggesting the strong ability of tumor targeting. Subsequently, the major organs and tumor tissue were obtained after mice euthanasia, and the results shown that the highest fluorescence intensity was observed in tumor tissue, followed by liver tissue, and no obvious aggregation of fluorescence signal was visible in the other tissues (Fig. 9A). These data suggested that the synthesized NMs proposed excellent tumor-targeting properties and presumably triggered the clearance of the reticuloendothelial system of the liver. In addition, as provided in Fig. 9B, no obvious body weight changes nor lethality of mice were monitored in the administration process of all groups, including P@FPP NMs treatment group. The H&E staining of major organs (liver, heart, lung, spleen, and kidney) was performed and the results indicated no appreciable organ damage nor noticeable abnormality (Fig. 9C). Thus, P@FPP NMs with superior tumor-targeting capability has low biological toxicity in vivo.

## Conclusions

Herbal extracts have shown sufficient potential in enhancing chemo-sensitivity, and the nano-herb is proposed and assembled to enhance the stability and tumor-targeting of herbal extracts. In this study, the synergistic effect of PRI in combination of PTX was confirmed using CCK-8 assay in A549 cells. Thus, a FA-modified active-targeting nano-herb micelles (denoted as P@FPP) were constructed for delivery of PRI and PTX to reverse the chemoresistance of NSCLC. The skeleton of PEG and grafted FA enhanced the tumor-targeting capacity and long plasma circulation of nano-herb, which also characterization of FTIR and ^1^H NMR, ζ-potential and size-distribution, and TEM. P@FPP exhibited robust inhibition of cell viability, migration, and invasion, while induction of cell apoptosis, which better than that of the PRI combined with PTX treatment. Moreover, the inhibition of tumor cell migration and invasion by P@FPP was associated with the EMT phenotypes, to some extent. P@FPP indicated excellent tumor-targeting capacity according to the in-vivo biodistribution analysis. In the animal experiments, P@FPP NMs revealed further inhibition of xenograft growth of mice and EMT phenotypes compared to the combination group, with low biotoxicity. In summary, this active-targeting P@FPP NMs realize tumor accumulation of nano-herb through “receptor-ligand interaction” with favorable biocompatibility, which showing great potential for clinical transformation.

## Supplementary Information

**Additional file 1: Fig. S1. **The peak area of ^1^H NMR was used to calculate the percentage of PTX in FA-PEG-PTX (left) and the standard absorbance curve of PRI and FA-PEG-PTX to calculate the drug-loading (right). **Fig. S2.** Cell viability evaluation of A549 cells treated with different concentrations of P@FPP NMs (n = 3). *: *P* < 0.05. **: *P* < 0.01. ***: *P* < 0.001. **Fig. S3.** A**–**B Wound healing and relative quantitative analysis of A549 cells with different treatments (n = 3). *: *P* < 0.05. **Fig. S4.** Jelly like substances of FA-PEG-COOH in the aqueous solution. **Table S1.** Primers using in this work for qRT-PCR analysis. **Table S2.** Primary antibodies used in this study for Western blot analysis.

## Data Availability

The datasets used and/or analyzed during the current study are available from the corresponding author on reasonable request.

## Abbreviations

EPR: Enhanced permeability and retention effect
EMT: Epithelial mesenchymal transformation
FA: Folic acid
FTIR: Fourier Transform Infrared Spectrogram
ICG: Indocyanine green
IC_50_: Half maximal inhibitory concentration
NMs: Nano-micelles
NSCLC: Non-small cell lung cancer
PEG: Polyethylene glycol
PRI: Pristimerin
PTX: Paclitaxel
TEM: Transmission electron microscopy
^1^H NMR: ^1^H nuclear magnetic resonance
