# Supplementary material for: Accurate delivery of pristimerin and paclitaxel by folic acid-linked nano-micelles for enhancing chemosensitivity in cancer therapy
Source: Nano Converg. 2022 Nov 24;9:52. doi: 10.1186/s40580-022-00343-5 (PMC9700544; doi:10.1186/s40580-022-00343-5)
Supplement: Supplementary file 1 — Additional file 1: Fig. S1. The peak area of 1H NMR was used to calculate the percentage of PTX in FA-PEG-PTX (left) and the standard absorbance curve of PRI and FA-PEG-PTX to calculate the drug-loading (right). Fig. S2. Cell viability evaluation of A549 cells treated with different concentrations of P@FPP NMs (n = 3). *: P < 0.05. **: P < 0.01. ***: P < 0.001. Fig. S3. A–B Wound healing and relative quantitative analysis of A549 cells with different treatments (n = 3). *: P < 0.05. Fig. S4. Jelly like substances of FA-PEG-COOH in the aqueous solution. Table S1. Primers using in this work for qRT-PCR analysis. Table S2. Primary antibodies used in this study for Western blot analysis. [file 40580_2022_343_MOESM1_ESM.docx]

Supporting information:

Accurate delivery of pristimerin and paclitaxel by folic acid-linked nano-micelles for enhancing chemosensitivity of non-small lung cancer cells

Chao Chen^1, #,^ *, Shiyu Du^1, #^, Wu Zhong^2, #^, Kunguo Liu^1^, Lihua Qu^3^, Feiyi Chu^2^, Jingjing Yang^1,^ *, Xin Han^1,^ *

^1^ Jiangsu Collaborative Innovation Center of Chinese Medicinal Resources Industrialization, School of Medicine & Holistic Integrative Medicine, Nanjing University of Chinese Medicine, Nanjing 210023, China.

^2^ Key Laboratory of Study and Discovery of Small Targeted Molecules of Hunan Province, School of Medicine, Hunan Normal University, Changsha 410013, China.

^3^ Department of Pathogenic Biology, School of Basic Medical Sciences, Wuhan University, Wuhan 430071, China.

* indicates the corresponding author

* Corresponding authors: Xin Han, Jingjing Yang, and Chao Chen

Tel./fax: 02585811382

E-mail address: Xin Han (xhan0220@njucm.edu.cn); Jingjing Yang (460183@njucm.edu.cn); Chao Chen (cchen22@njucm.edu.cn)

# Chao Chen, Shiyu Du, and Wu Zhong contribute equally to this work.

1. Materials

NH_2_-PEG-COOH (2000 kDa) was from Shanghai Macklin Biochemical Co., Ltd (Shanghai, China). Folic acid (FA) and Paclitaxel (PTX) were from Aladdin (Shanghai, China). Pristimerin (PRI) was purchased from Yuanye Bio-Technology (Shanghai, China).

2. Methods

2.1. Cytotoxicity evaluation of P@FPP NMs

A549 cells (4 × 10^4^ cells/well) were seeded into 96-well plate and incubated for 18-24 hours and then administrated with different concentrations of P@FPP NMs (0-200 μg/mL) for 48 hours. After that, 10 µL of CCK8 reagents (YIFEIXUE BIO TECH) were added and incubated for 60 minutes, then the optical density at 450 nm was examined by a Multimode Plate Reader.

2.2. Wound healing assay

The A549 cells were seeded into 6-well plates at a density of 2 × 10^5^ cells/mL. After the cells were adhered for 18-24 hours, a straight scratch was made on the cell monolayer using a sterile 100 µL pipette tip, then the culture medium was refreshed to the serum-free DMEM and incubated with free-drugs or P@FPP nano-micelles for 48 hours. After that, ImageJ 1.52a software (National institutes of Health, USA) was used to measure wound closure area.


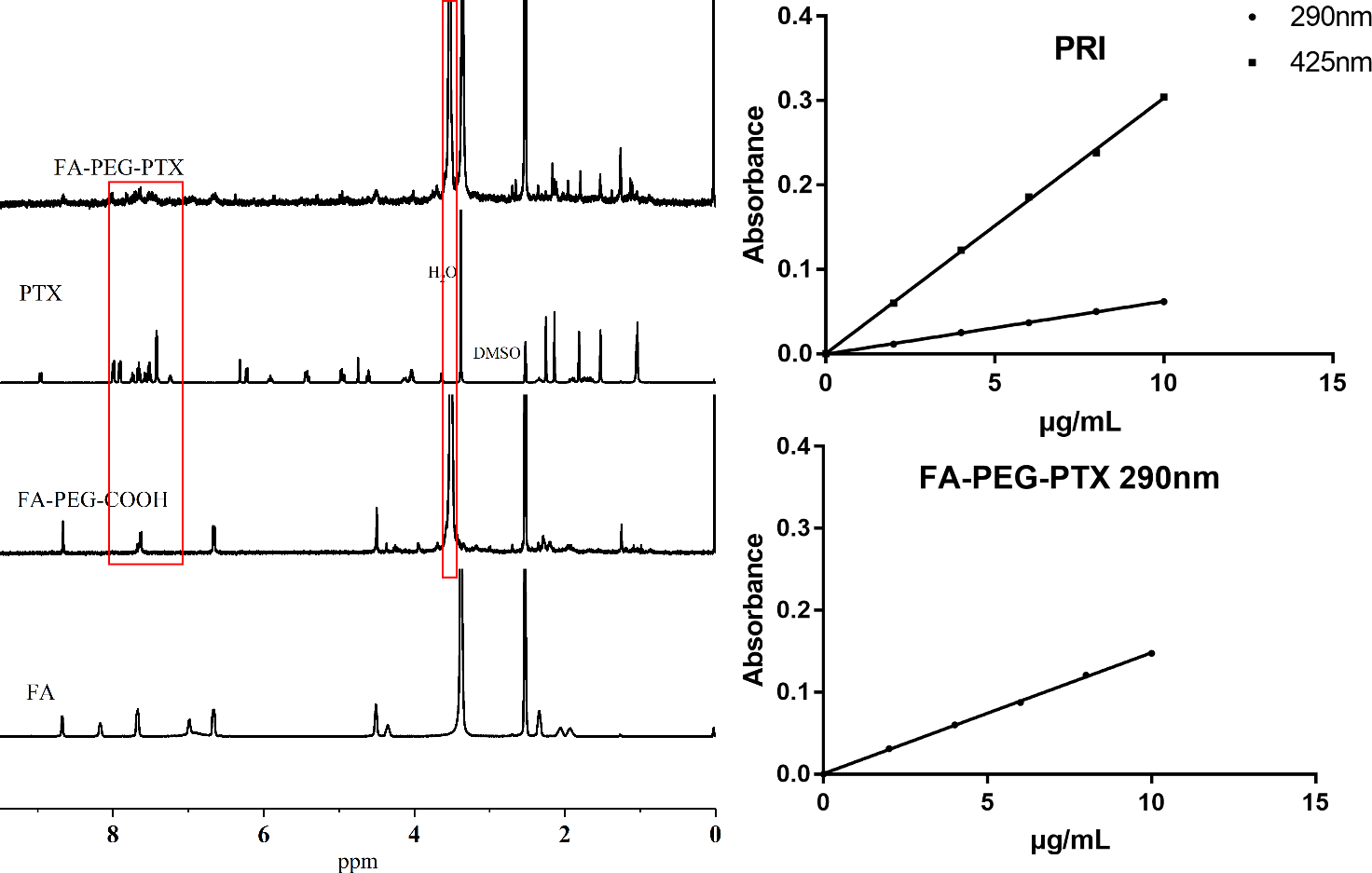


**Fig. S1.** The peak area of ^1^H NMR was used to calculate the percentage of PTX in FA-PEG-PTX (left) and the standard absorbance curve of PRI and FA-PEG-PTX to calculate the drug-loading (right).


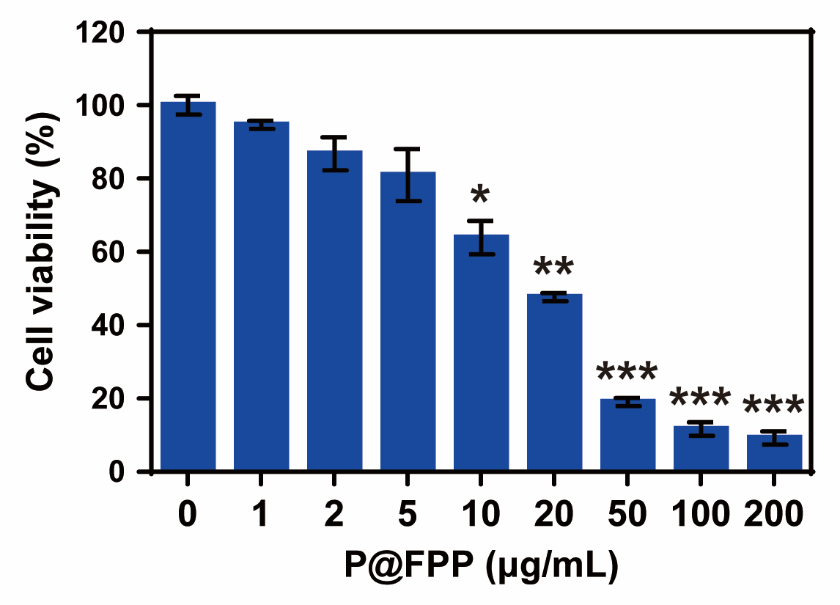


**Fig. S2.** Cell viability evaluation of A549 cells treated with different concentrations of P@FPP NMs (n = 3). *: *P* < 0.05. **: *P* < 0.01. ***: *P* < 0.001.


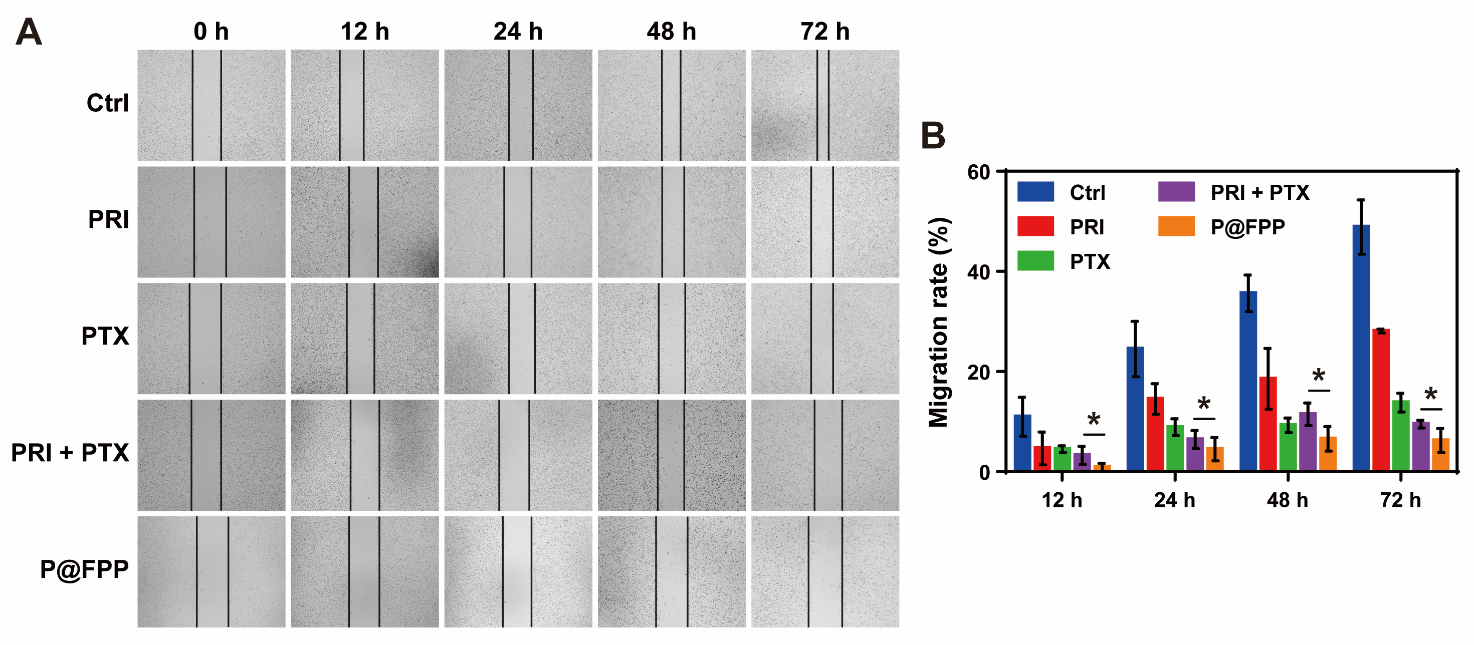


**Fig. S3.** **A-B** Wound healing and relative quantitative analysis of A549 cells with different treatments (n = 3). *: *P* < 0.05.

**
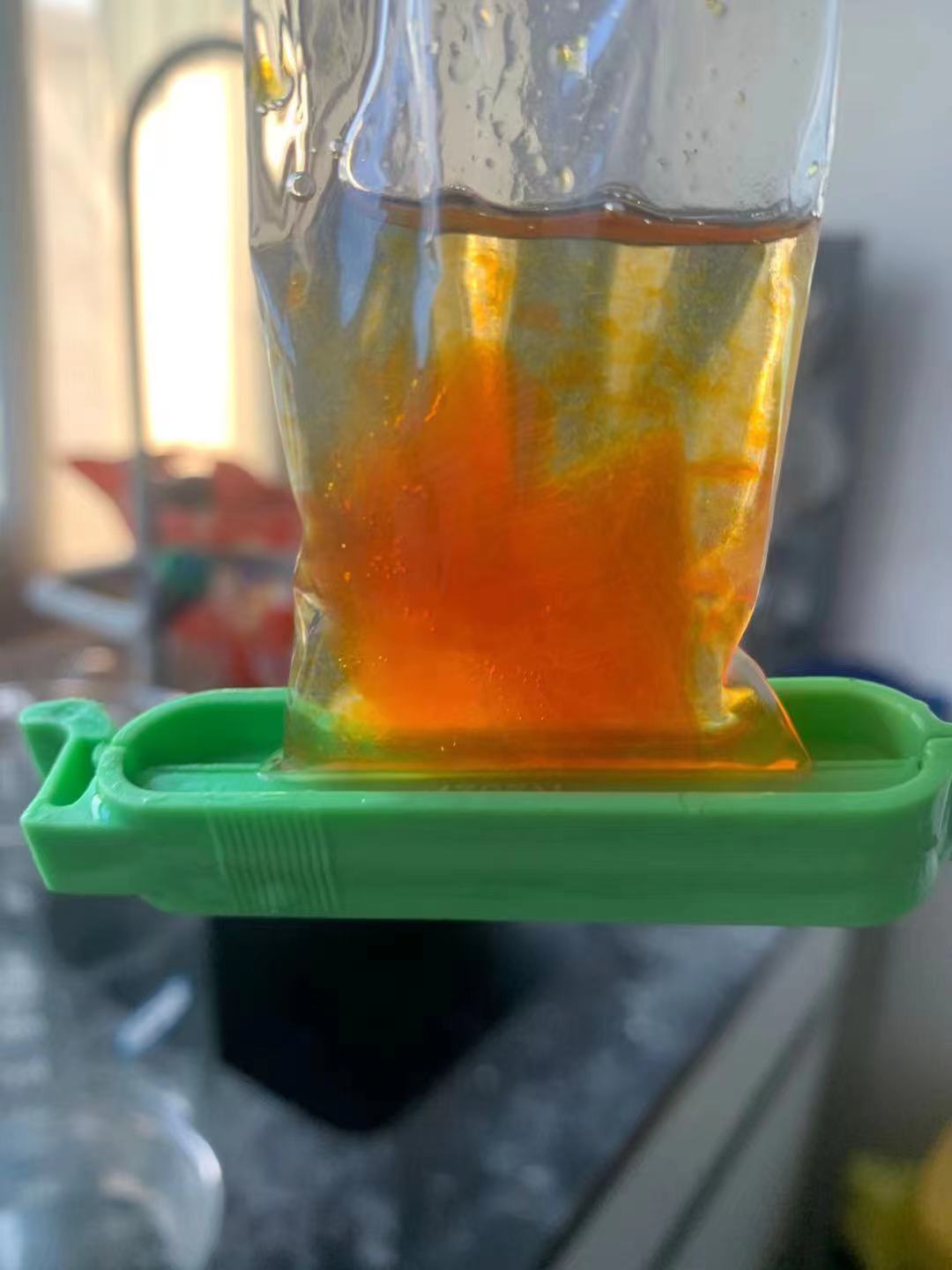
**

**Fig. S4.** Jelly like substances of FA-PEG-COOH in the aqueous solution.

Table S1. Primers using in this work for qRT-PCR analysis.

| **Gene** | **Primer sequence (5′-3′)** |
| --- | --- |
| E-cadherin | Forward: CGAGAGCTACACGTTCACGG  Reverse: GGGTGTCGAGGGAAAAATAGG |
| N-cadherin | Forward: TCAGGCGTCTGTAGAGGCTT  Reverse: ATGCACATCCTTCGATAAGACTG |
| Vimentin | Forward: CGTCCACACGCACCTACAG  Reverse: GGGGGATGAGGAATAGAGGCT |
| Twist | Forward: GGACAAGCTGAGCAAGATTCA  Reverse: CGGAGAAGGCGTAGCTGAG |
| β-actin | Forward: GTGCTATGTTGCTCTAGACTTCG  Reverse: ATGCCACAGGATTCCATACC |

Table S2. Primary antibodies used in this study for Western blot analysis.

| **Antibodies** | **Code** | **Company** |
| --- | --- | --- |
| E-cadherin | 3195S | Cell Signaling Technology |
| Vimentin | 5741S | Cell Signaling Technology |
| N-cadherin | 610920 | BD Biosciences-US |
| GAPDH | AP0066 | Bioworld |
